#  Evaluation of Bactereuria and Antimicrobial Susceptibility among Hospitalized Patients With and Without Catheter in Kerman Province- Iran in 2011 

**Published:** 2013

**Authors:** Farhad Sarrafzadeh, Seyed Mojtaba Sohrevardi

**Affiliations:** a *Neuroscience Research Center and Faculty of Medicine, Kerman University of Medical Sciences, Kerman, Iran. *; b *Faculty of Pharmacy, Shahid Sadoughi University of Medical Sciences, Yazd, Iran. *

**Keywords:** Nosocomial, Infection, UTIs, Urinary catheter, *E.coli*, Antibiotic

## Abstract

Nearly 15% to 25% of patients in general hospitals have a catheter at some time during their stay. Up to 97% of nosocomial urinary tract infections (UTIs) are related to catheter. The type of bacteremia is usually polymicrobial which makes the treatment more difficult. Previous studies showed an increase in mortality from bacteremia in these patients. The aim of the present study was to compare the prevalence of UTIs among patients with and without catheter, and to detect the type of bacteriuria and antibacterial resistance pattern.

In this cross sectional study, samples were taken between Jan 2011 and July 2011. 678 hospitalized patients in different wards of Afzalipour hospital, Kerman- Iran, were enrolled in the study. E-test was applied to detect the pattern of resistance to gentamicin, nitrofurantoin, ciprofloxacin, ceftriaxon and co-trimoxazole. Results showed positive culture samples in 86% of female patients.

*Escherichia coli, Candida *and *Staphylococcus aureus *were detected in 72, 20 and 7 percent of the positive cultures, respectively. 52.3% of detected *E.coli *was sensitive to gentamicin , 62% to ceftriaxone, 71.4% to ciprofloxacin, and 91.9% were sensitive to nitrofurantoin. Therefore, the most sensitive antibiotics in UTIs were ciprofloxacin and *ceftriaxone*. Unfortunately, the rate of antibacterial drug resistance was high in comparison with developed countries. Wise selection of antibiotics at hospitals and increasing the knowledge of patients to prevent self use of antibiotics can reduce antimicrobial resistance.

## Introduction

The treatment of nosocomial infections is a costly procedure. Urinary tract infections (UTIs) account for 40-45% of hospital infections and cause spread of antibiotic resistant bacteria resources at the hospitals (1). Nearly 80% of hospital-acquired UTIs are associated with permanent catheter (2). Urinary catheter infections are common causes of nosocomial UTIs. Due to prevention of complete emptying of bladder, catheters can reduce the host’s natural defense and provide the environment for bacterial growth (3). Inserting a permanent catheter into the urethra in a hospitalized patient makes easy access to a sterilized location for pathogens (2). Up to 97% of nosocomial UTIs are associated with insertion of instruments, including catheter, into urinary tract. (4, 5), and annually account for up to 40% of nosocomial infections in the US hospitals (6- 8). UTIs often cause infection in long term care facilities (LTCFs) and most of them are catheter-associated (9, 10). Using catheter in the urinary tract is very prevalent in hospitals and LTCFs and seems to be increasing, at least in hospitals (11). Nearly 15% to 25% of patients in general hospitals have a catheter at some time during their stay (7, 12, and 13). Urethral catheterization manages nearly 5%to 10% of LTCFs residents (10, 13, 14), most of the time for bladder outlet block in men and urine incontinence of women (12, 15, 16). The period of catheterization is the most significant risk factor for development of catheter-associated bacteriuria (13, 17, 18). Nosocomial UTIs are often the most popular source of bacteremia in LTCFs, and the type of the bacteremia is usually polymicrobial in these patients (19, 20), which makes the treatment more difficult. Studies show that in these patients, mortality of bacteremia is three time more probable (21). Despite the limited and foreseeable spectrum of the causing agents in uncomplicated UTIs, a wide range of bactreria can cause nosocomial UTIs, and many are resistant to numerous antimicrobial drugs (22, 23). One of the most important problems in treating infectious diseases is lack of sufficient information from microorganism’s resistance pattern to antibiotics. The study conducted in 1997 in Bangladesh reported that the microbial resistance to co-trimoxazole was 43% (24). The other study in Qazvin-Iran in 1999 showed that *Klebsiella pneumonia *was the most common bacterial source related to catheter in genecology ward (25). The other study in 1997 showed the highest prevalence of microbial mass was related to the *Escherichia coli *(26). In 1998 in Tehran, the greatest resistance to usual antibiotics in urinary tract infections was co-trimoxazole (78.1%) (27). Since there is no sufficient data regarding antibiotic resistance to UTIs in Iran, we conducted this study in order to achieve practical patterns in treatment of infections by considering the amount and type of the bacteriuria and making decision to choose effective antibiotics in patients with and without catheter in our region. We hope that the information obtained in this research help us to find the way to reduce the costs of treatment and the period of hospitalization. 

## Experimental

In this cross-sectional study, samples were taken between Jan 2011 and July 2011 from all patients in different wards of Afzali-Pour general hospital, Kerman-Iran. 678 cases were enrolled in the study. According to having or not having the catheter, sampling methods were different. In patients without catheter, the middle part of the first morning urine sample were used and collected in disposable containers (midstream method). In patients who had urine catheter, samples were taken after catheter clamping, disinfecting outer part of the catheter and entering sterile syringes to the upper part of the catheter. Finally, urine samples were collected in 578 patients (85.63%) by midstream method and in 100 patients (14.37%) through the catheter. Samples were immediately transported to the laboratory and cultured on the blood agar environment (for differentiate types of *Enterobacterias *family). After 24 h incubation at 37°C, colonies were counted. Samples with colonies counted more than 10^5^ were considered as positive (21). E-test strips (*Biomerieux, Sweden) *were used for antibiogram. This test uses a strip coated with a logarithmic gradient of an antimicrobial agent applied to an inoculated plate. After incubation, an ellipse of inhibition was formed. At the intersection of the ellipse with the strip, the MIC (minimum inhibitory concentration) was read from the interpretive scale and was converted to three sensitivity classes (sensitive, semi-sensitive, resistant) according to the breakpoints of Clinical and Laboratory Standards Institute (28). SPSS-16 was used for statistical analysis. Fisher’s exact test was used to determine the statistical differences for *E. coli *frequencies between patients with and without catheter.

## Results

Patients were divided into two groups; with and without catheter. Among 578 patients without catheter, 53.8% were female, 59.4% were married and 42.3% had a history of other disease. In 100 patients who had catheter, 50% were female, 71% were married and 67% had a history of other disease. Out of 29 positive culture patients, 25 patients were female. Table 1 shows the frequency of UTI causing agents in patients. 649 out of 678 cases had negative cultures and only 4.3% of the total hospitalized patients had UTIs based on urine culture results. 

**Table 1:** Frequency of infection causing agents among patients

		Negative culture	E.coli	Staphylococcus coagulase negative	Streptococ hemoliti	Candida	Klebsiella	Pseudomonas	Total
Without catheter	Number	560	14	2	1	1	0	0	578
	Percent	96.88%	2.43%	0.35%	0.17%	0.17%	0%	0%	100%
With catheter	Number	89	7	0	0	2	1	1	100
	Percent	89%	7%	0%	0%	2%	1%	1%	100%
Total	Number	649	21	2	1	3	1	1	678
	Percent	95.72%	3.1%	0.3%	0.15%	0.44%	0.15%	0.15%	100%
	p-value		0.024						

Eleven percent of catheterized patients and 3.2% of patients without catheter had urine positive culture. Our study shows that *E. coli *was the leading cause of infection among our patients (72%). Frequency of *E coli *infection in catheter-associated patients was lower than patients without catheter (p = 0.024). *Candida *(10%) and *Staphylococcus aureus *(7%) were other important infection causing agents. 

Tables 2 shows *E.coli *susceptibility to nitrofurantoin. 61.9% of the cases were sensitive and 9.5% of them were resistant to nitrofurantoin. Most of the patients who were sensitive to nitrofurantoin did not have catheter. 

**Table 2:** Pattern of E.coli resistance to nitrofurantoin

		**Sensitive **			**Semi-sensitive **		**Resistant **		**Total **	
		**Number **	**Percent **	**Number**	**Percent **	**Number **	**Percent **	**Number **	**Percent **
**Without catheter **			10	71.4%	3	%21.4	1	%7.2	14	100%
**With catheter **			3	42.8%	3	42.8%	1	14.4%	7	100%
**Total **			13	61.9%	6	28.6%	2	9.5%	21	100%
**MIC (mcg/mL) **			21		45		138			

Table 3 shows that 52.3% of the cases were sensitive and 9.6% of them were resistant to gentamicin. Among the patients sensitive to gentamicin, 4 cases had catheter and 7 patients were without catheter. Table 4 shows that 62% of the patients were sensitive and 19% of them were resistant to ceftriaxon. Table 5 shows that totally, 71.4% of the patients were sensitive, and 14.3% were resistant to ciprofloxacin. More details have been shown in the tables. 

Nonetheless, no significant difference was observed in the efficacy of the antibiotics in diabetic, renal and hypertensive patients (p > 0.05). 

**Table 3:** Pattern of E.coli resistance to gentamicin

		**Sensitive **		**Semi-sensitive **		**Resistant **		**Total**	
		**Number **		**Percent **	**Number **	**Percent **	**Number **	**Percent **	**Number **	**Percent **
**Without catheter **	7		50%	5	35.7%	2	14.3%	14	100%
**With catheter **	4		57%	3	43%	0	0%	7	100%
**Total **	11		52.3%	8	38.1%	2	9.6%	21	100%
**MIC (mcg/mL) **	2			7		19			

**Table 4:** Pattern of E.coli resistance to ceftriaxon

	**Sensitive **		**Semi-sensitive **		**Resistant **		**Total **	
	**Number **	**Percent **	**Number **	**Percent **	**Number **	**Percent **	**Number **	**Percent **
Without catheter	9	64.3%	3	21.4%	2	14.3%	14	100%
With catheter	4	57.2%	1	12.3%	2	28.5%	7	100%
Total	13	62%	4	19%	4	19%	21	100%
MIC (mcg/mL)	5		27		76			

**Table 5:** Pattern of E.coli resistance to ciprofloxacin

		**Sensitive **		**Semi-sensitive **		**Resistant **		**Total **	
	**Number **		**Percent **	**Number **	**Percent **	**Number **	**Percent **	**Number **	**Percent **
**Without catheter **	10		71.4%	2	14.3%	2	14.3%	14	100%
**With catheter **	5		71.4%	1	14.3%	1	14.3%	7	100%
**Total **	15		71.4%	3	14.3%	3	14.3%	21	100%
**MIC (mcg/mL) **	0.8			1.4		5.6			

## Discussion

In this study, for the first time, antimicrobial susceptibility was compared between hospitalized patients with or without urinary catheter. Most of the previous studies regarding antimicrobial resistance were performed using disk diffusion method that is old and almost non sensitive. We used E-test strip that is more sensitive and could show MIC for different antimicrobial agents. In this study, the frequency of catheterization between women and men was the same, but the rate of catheterization in married patients was significantly more than single patients. There was no significant difference between the gender of the patients who were involved the study. In catheterized patients, 50% and in patients without catheter 53% were female. As the previous study showed (29), women (86%) are more susceptible to UTIs in comparison to men in our study. 

Surprisingly, only 4.3% of the hospitalized patients were culture positive. possibly because most of our patients took antibiotic before admission to the hospital. Almost all of the Antibiotics (oral or parenteral) can bought easily and without a prescription from pharmacies around the city. These drugs are inexpensive, and most of the patients prefer to do self-medication once they got symptoms of infection.

This study showed that the most sensitive antibiotic for *E.coli *in patients with underlying cardiovascular disease and also in patients with diabetes was gentamicin, however, the antibiotic of choice in patients with renal disease was ciprofloxacin. In this study, the most common cause of urinary tract infections was *E.coli*, and ciprofloxacin was also a good choice because of the low microbial resistance compared with other evaluated antibiotics.

In the study conducted in Brazil, *E.coli *(26%), *Pseudomonas aeruginosa *(15%) and *Klebsiella pneumonia *(11%) were the main causes of UTIs. In that research, the rate of *E.coli *sensitivity was 93% to *ceftriaxone *and 78% to ciprofloxacin.(30). However, in our study *E.coli *was detected in 72% of the patients, and the sensitivity to ciprofloxacin (71.4%) was interestingly higher than *ceftriaxone *(62%). It may be related to misuse of ceftriaxon in our community which is because of patients’ desire for injectable medicine, emphasis on prescribing injectable drugs, and cultural problem and misleading information about the efficacy of injectable medicine in comparison with oral medications. Researches in two hospitals in Kuwait during one year have shown that the most common microbial masses were *E.coli *(47%), *Candida *(10%) and *Klebsiella pneumoniae *(9.6%) (31). A research in Medical College Hospital in India showed that the most common bacterial causes of UTIs were *Staphylococcus aureus *( 27%) and *E.coli *(21%) [26], but our results were different. Previous study conducted in 2000 in Kerman showed that *E.coli *was the microbial cause of UTIs in 63% of patients. *E.coli *susceptibility was around 69% to nitrofurantoin, 92% to ciprofloxacin, 37% to gentamicin and 38% to co-trimoxazole (32). Whereas in our study and after 11 years, *E.coli *sensitivity to gentamicin increased by 52.3%. It may be because of introducing ceftriaxon to market and over-prescribing of the drug. It seems that gentamicin was replaced with ceftriaxon here in patients who need injectable medicine for UTIs. However, ciprofloxacin sensitivity had decreased from 92% to 71.4%. In a research performed in the gynecological department of Qazvin-Iran University hospital, urine samples taken from146 catheter-associated patients showed the following microbial pattern: *Klebsiella pneumoniae *60%, *E.coli *25%, *Proteus mirabilis *5%, *Pseudomonas aeruginosa *2.5% (25). Studies in Bangladesh in 1997 showed that the microorganism sensitivity to ciprofloxacin was74% and this drug was more active than other antibiotics against Gram-negative bacteria (24) which is consistent with our results In the other study in the ICU of Afzalipour hospital, the most common identified microorganisms were *Klebsiella *(90.6%), *Acinetobacter *(28.1%) and *Pseudomonas *(21.9%) (33). It was concluded that ICU can have different microbial frequency from general wards. As regarded in this research, the most sensitive antibiotics in UTIs are ciprofloxacin and *ceftriaxone*. They are broad-spectrum antibiotics, and irrational use of them leads to an increase in the rate of microbial resistance not only in UTIs, but also in all the infectious disease. However, irrational use of ceftriaxon in our country resulted in 60 patient’s death. Hence, practitioner must be aware of the slow infusion and incompatibility with calcium ions (34). Our results showed that catheter associated patients were more resistant to nitrofurantoin and ceftriaxon. Drug and therapeutic committees (DTC) can help medical staff to choose antibiotics wisely in each field based on the guidelines or protocols. Standard hospital pharmacy unit and hospital or clinical pharmacists can prevent the development of antibiotic resistance by preparing injectable medicine under aseptic condition, and helping physicians choose the proper antibiotics based on patients sample culture results and avoiding prolonged catheterization. 

## References

[1] Longo DL, Fauci AS, Kasper DK, Hauser SL, Jameson JL, Loscalzo J (2012). Harrison's Principles of Internal Medicine.

[2] Andreoli TE, Benjamin I, Griggs RC, Wing EJ (2010). Cecil Essentials of Medicine.

[3] Marcdante KJ, Kliegman RM, Jenson HB, Behrman RE (2011). Nelson Essentials of Pediatrics.

[4] Bronsema DA, Adams JR, Pallares R (1993). Secular trends in rates and etiology of nosocomial urinary tract infections at a university hospital. J. Urol.

[5] Richards MJ, Edwards JR, Culver DH, Gaynes RP (2000). Nosocomial infections in combined medical surgical intensive care units in the United States. Infect. Control Hosp. Epidemiol.

[6] Tambyah PA (2004). Catheter associated urinary tract infections: diagnosis and prophylaxis. Int. J. Antimirob. Agents.

[7] Haley RW, Hooton TM, Culver DH, Stanley RC, Emori TG, Hardison CD, Quade D, Shachtman RH, Schaberg DR, Shah BV, Schatz GD (1981). Nosocomial infections in U.S. hospitals, 1975-1976: estimated frequency by selected characteristics of patients. Am. J. Med.

[8] Haley RW, Culver DH, White JW, Morgan WM, Emori TG (1985). The nationwide nosocomial infection rate. A new need for vital statistics. Am. J. Epidemiol..

[9] Nicolle LE, Strausbaugh LJ, Garibaldi RA (1996). Infections and antibiotic resistance in nursing homes. Clin. Microbiol. Rev.

[10] Smith PW, Bennett G, Bradley S, Drinka P, Lautenbach E, Marx J, Mody L, Nicolle L, Stevenson K (2008). SHEA/ APIC guideline: infection prevention and control in the long- term care facility,July 2008. Infect. Control Hosp. Epidemiol.

[11] Weinstein JW, Mazon D, Pantelick E, Reagan- Cirincione P, Dembry LM, Hierholzer WJ Jr (1999). A decade of prevalence surveys in a tertiary care center:trends in nosocomial infection rates,device utilization, and patient acuity. Infect. Control Hosp. Epidemiol.

[12] Garibaldi RA, Burke JP, Dickman ML, Smith CB (1974). Factors predisposing to bacteriuria during indwelling urethral catheterization. N. Engl. J. Med.

[13] Warren JW (1997). Catheter associated urinary tract infections. Infect. Dis. Clin. North. Am.

[14] Warren JW (1994). Catheter associated bacteriuria in long term care facilities. Infect. Control Hosp. Epidemiol.

[15] Kunin CM, McCormark RC (1966). Prevention of catheter induced urinary tract infections by sterile closed drainage. N. Eng. J. Med.

[16] Classen DC, Larsen RA, Burke JP, Stevens LE (1991). Prevention of catheter- associated bacteriuria: clinical trial of methods to block three known pathways of infection. Am. J. Infect. Control.

[17] Sait S, Lipsky BA (1999). Preventing catheter related bacteriuria: should we? Can we? How?. Arch. Intern. Med.

[18] Maki DG, Tambyah PA (2001). Engineering out the risk for infection with urinary catheters. Emerg. Infect. Dis.

[19] Muder RR, Brennen C, Wagener MM, Goetz AM (1992). Bacteremia in a long- term care facility:a five year prospective study of 163 consecutive episodes. Clin. Inect. Dis.

[20] Rudman D, Hontanosas A, Cohen Z, Mattson DE (1988). Clinical correlates of bacteremia in a veterans administration extended care facility. J. Am. Geriatr. Soc.

[21] Platt R, Polk BF, Murdock B, Rosner B (1981). Mortality associated with nosocomial urinary tract infection. N. Engl. J. Med.

[22] Nicolle LE (2005). Catheter related urinary tract infection. Drugs Aging.

[23] Hooton TM, Stamm WE (1997). Diagnosis and treatment of uncomplicated urinary tract infection. Infect. Dis. Clin. North. Am.

[24] Iqbal, Rahman M, Kabir MS (1997). Increasing ciprofloxacin resistance among prevalent urinary tract bacterial isolates in Bangladesh. Jpn. J. Med. Sci. Biol.

[25] Sharifi M (1999). Bacteriuria in catheterized patients of gynecology ward. Arc. Iranian Med.

[26] Chaudhari R, Deshpande, Angadi SA, Koppikar GV A prielimirary study of catheter associated bacteriuria. Hospital J.

[27] Norouzi J, Mirjalili A, Ajdari A (2000). Evaluation of urinary tract infections among disabledandelderly Kahrizak-Tehran rest home. Feyz.

[28] Clinical and Laboratory Standards Institute (1990). Approved standard M7-A2. Methods forDilution Antimicrobial Susceptibility Tests for Bacteria that Grow Aerobically.

[29] Dromigny JA, Ndoye B, Macondo EA (2003). Increasing prevalence of antimicrobial resistance among Enterobacteriaceae uropathogens in Dakar, Senegal: a multicenter study. Diagn. Microbiol. Infect. Dis.

[30] Neto JAD, da Silva LDM, Martins ACP, Tiraboschi RB, Domingos ALA, Suaid HJ, Tucci S, Cologna AJ (2003). Prevalence and bacterial susceptibility of hospital acquired urinary tract infection. Acta Cirurgica Brasileira.

[31] Sweih NA, Jamal W, Rotimi VO (2005). Spectrum and antibiotic resistance of pathogens isolated from hospital and community patients with urinary tract infections in two large hospitals in Kuwait. Med. Principl. Prac.

[32] Sepehri GR, Dabiri S, Vosoogh MR (2004). Comparison the sensitivity of microbial agents causing urinary tract infections to commonly used antibiotics in Kerman in the years 1995 and 2000. J. Rafsanjan Univ. Med. Sci.

[33] Sarafzadeh F, Sohrevardi SM, Garehghozli M, Ahmadinejad M (2010). Detection of the most common microorganisms and their resistance against anti-microbials in intubated patients in an ICU in Kerman, Iran. Iranian J. Pharm. Res.

[34] Iran Food (2009). Drug Organization. Lethal adverse effects related to ceftriaxon. Letter No. 102.

